# Preinoculation of Soybean Seeds Treated with Agrichemicals up to 30 Days before Sowing: Technological Innovation for Large-Scale Agriculture

**DOI:** 10.1155/2017/5914786

**Published:** 2017-10-10

**Authors:** Ricardo Silva Araujo, Sonia Purin da Cruz, Edson Luiz Souchie, Thomas Newton Martin, André Shigueyoshi Nakatani, Marco Antonio Nogueira, Mariangela Hungria

**Affiliations:** ^1^Total Biotecnologia Indústria e Comércio S/A, Rua Emilio Romani, No. 1190, CIC, Curitiba, PR, Brazil; ^2^Universidade Federal de Santa Catarina, Campus Curitibanos, Rodovia Ulisses Gaboardi, Km 3, Curitibanos, SC, Brazil; ^3^Instituto Federal Goiano, Campus Rio Verde, Rodovia Sul Goiana, Km 01, Caixa Postal 66, Rio Verde, GO, Brazil; ^4^Universidade Federal de Santa Maria, Avenida Roraima, No. 1000, Santa Maria, RS, Brazil; ^5^Embrapa Soja, CP 231, 86001-970 Londrina, PR, Brazil

## Abstract

The cultivation of soybean in Brazil experienced an expressive growth in the last decades. Soybean is highly demanding on nitrogen (N) that must come from fertilizers or from biological fixation. The N supply to the soybean crop in Brazil relies on the inoculation with elite strains of* Bradyrhizobium japonicum, B. elkanii, *and* B. diazoefficiens*, which are able to fulfill the crop's N requirements and enrich the soil for the following crop. The effectiveness of the association between N_2_-fixing bacteria and soybean plants depends on the efficacy of the inoculation process. Seed treatment with pesticides, especially fungicides or micronutrients, may rapidly kill the inoculated bacteria, affecting the establishment and outcome of the symbiosis. The development of technologies that allow inoculation to become a successful component of industrial seed treatment represents a valuable tool for the seed industry, as well as for the soybean crop worldwide. In this article, we report the results of new technologies, developed by the company Total Biotecnologia Indústria e Comércio S/A of Brazil, for preinoculation of soybean seeds with bradyrhizobia, in the presence of agrichemicals. Our results demonstrate improved bacterial survival for up to 30 days after inoculation, without compromising nodulation, N_2_-fixation, and yield in the field.

## 1. Introduction

The cultivation of soybean [*Glycine max *(L.) Merr.] in Brazil is one of the economic activities that experienced the most expressive continuous growth in the last decades [1]. Brazil is currently one of the major producers of soybean in the world, along with the United States of America [2]. The crop occupied around 33 million hectares in the 2015/2016 cropping season, with a total grain production of over 95 million tons, and the grain is the leading commodity of the Brazilian agribusiness, accounting for approximately 13% of all Brazilian exportations [3, 4]. The success of the soybean crop in Brazil, however, could have never been achieved without the full utilization of the natural process of symbiotic nitrogen (N_2_) fixation [5].

Soybean is highly demanding on nitrogen (N), requiring around 80 kg of N to produce 1,000 kg of grains [6]. Considering an average grain yield of 3,000 kg ha^−1^, in Brazil, and 33 million hectares grown with soybean, an astounding 7.9 × 10^6^ tons of N (equivalent to 16.5 × 10^6^ tons of urea) would be necessary to fulfill the crop's demand for N. If all that N was to be supplied by chemical fertilizers, whose efficiency of utilization reaches, at most, 50%, an estimate of 33 million tons of urea would be necessary, and the costs of N fertilization alone would render the soybean crop completely unattractive under Brazilian conditions. However, the cultivation of soybean in Brazil relies on the inoculation with elite strains of* Bradyrhizobium japonicum, B. elkanii, *and* B. diazoefficiens*, which are able to supply all the soybean N requirements and enrich the soil for the following crop [5, 6]. This technology generates an economy of over 15 billion US dollars that would have to be spent with chemical fertilizers each season to supply the crop's demands for N [5].

The effectiveness of the association between N_2_-fixing bacteria and soybean plants depends on the efficacy of the inoculation process, which consists of coating the seed surface with either solid (peat-based) or liquid bacterial formulations. Many factors may negatively affect the survival of the bacteria after seed inoculation [7, 8], resulting in failures in the nodulation process and, consequently, in N_2_ fixation and N supply to the crop. Seed treatment with pesticides, especially fungicides [9–13] or micronutrients [14], may rapidly kill the inoculated bacteria, affecting the establishment and outcome of the symbiosis [15].

In-furrow inoculation has long been postulated as an alternative to traditional seed inoculation in order to avoid damage to the bacteria [17, 18]. After the liquid inoculant formulations have become a consolidated technology, in-furrow inoculation has been successfully employed for soybean [8, 19]. In addition, spray inoculation has been shown to be effective as remedial inoculation, in cases of failures of soybean nodulation [8]. Nevertheless, the most commonly adopted and practical form of inoculation is still on the seeds.

The increasing technological level of agricultural activities, as well as the high cost of labor, along with issues concerning human safety in the manipulation of pesticides, however, has made on-farm seed manipulation unattractive. Industrial seed treatment that combines the application of pesticides, micronutrients, stimulants, and other additives, as well as inoculants, to the seeds, prior to delivery to the farmer is becoming popular [20]. However, such practices may be very deleterious to the inoculated bacteria and, in most cases, bacterial mortality is so high that no viable bacteria are present on the seeds at sowing [21]. The development of technologies that allow inoculation to become a successful component of industrial seed treatment represents a valuable tool for the seed industry, as well as for the soybean crop worldwide. In this article, we report the results of new technologies, developed by the company Total Biotecnologia Indústria e Comércio S/A of Brazil, for preinoculation of soybean seeds with bradyrhizobia, in the presence of agrichemicals. Our results demonstrate improved bacterial survival for up to 30 days after inoculation, without compromising nodulation, N_2_-fixation, and yield in the field.

## 2. Materials and Methods

### 2.1. Inoculants and Technologies Employed in This Study

All inoculants employed in this study were commercial products manufactured and commercialized by Total Biotecnologia Indústria e Comércio S/A, in Curitiba, State of Paraná, Brazil. They contain the strains SEMIA5079* (Bradyrhizobium japonicum)* and SEMIA5080* (B. diazoefficiens)*, which are officially authorized for the composition of commercial inoculants for soybean in Brazil. TotalNitro™ is a standard liquid inoculant [5 × 10^9^ colony forming units (CFU) mL^−1^] and was employed without any additives or seed treatment chemicals, as a control in some experiments. Standard peat-based TotalNitro inoculant (5 × 10^9^ CFU g^−1^) was employed either with or without chemicals as a second control in some experiments. Cronos™ technology [liquid inoculant (5 × 10^9^ CFU mL^−1^), along with two additives (A and B)] was employed in a 2-day preinoculation scheme in the presence of the chemical (insecticide/fungicide) Standak Top™ (Fipronil + Pyraclostrobin + Methyl thiophanate), and in a 4-day preinoculation scheme in the absence of the chemical. CronosNod™ Technology [liquid inoculant (7 × 10^9^ CFU mL^−1^), along with CronosNod protective additive], was employed in a 7-day preinoculation scheme in the presence of the chemical Standak Top. Finally, CronosTSI™ Technology [standard peat-based TotalNitro inoculant (5 × 10^9^ CFU g^−1^) along with two TSI™ additives, A and B] was employed in a 30-day preinoculation scheme, also in the presence of the chemical Standak Top. All additives employed are bacterial protectors composed of sugars, polymers, and inert substances that exert osmoprotection of the bacteria and potentiate inoculation.

### 2.2. Laboratory Experiments

Laboratory experiments performed at Embrapa Soja, Londrina, State of Paraná, and at the Instituto Federal Goiano, Rio Verde, State of Goiás, compared the survival of bradyrhizobia on the surface of seeds that had been inoculated with the preinoculation technologies evaluated in this study, in the presence of chemicals, with standard inoculants on untreated seeds. Bacterial survival was estimated as the number of CFU recovered from the surface of inoculated seeds at different times after inoculation. Treatments depended on the institution that performed the tests and were (1) standard liquid (TotalNitro) inoculant, applied to untreated seeds without additives 2 h before evaluation (time zero); (2) Cronos Technology, applied to treated seeds 2 h and 2 and 4 days before evaluation; (3) CronosNod Technology, applied to treated seeds 2 h and 2, 4, 7, 14, and 21 days before evaluation; and (4) CronosTSI Technology, applied to seeds 2 h and 2, 4, 7, 14, 21, 28, and 35 days before evaluation.

For seed treatment and inoculation, all products were applied to the seeds according to their manufacturers' instructions regarding doses and methods of application. In case chemicals were used, they were applied to the seeds prior to inoculation with bradyrhizobia. Sample lots of 1 kg of certified seeds were inoculated for each treatment. Cultivar BMX Potência RR and NS 7209 Pro were employed at Embrapa Soja and at the Instituto Federal Goiano, respectively. After treatment and inoculation, seeds were stored under controlled laboratory conditions, at 60% relative humidity and 25°C ± 2°C.

The recovery of bacterial cells from inoculated seeds was quantified with the methodology established by Brazilian regulations [22]. At the appropriate sampling times, four subsamples of 100 seeds each were withdrawn from each treatment. Each 100-seed subsample was aseptically transferred to a 250-mL Erlenmeyer flask containing 100 mL sterile 0.85% (w/v) NaCl + 0.01% (w/v) Tween 80 solution, in order to obtain the 10^0^ dilution. Flasks were then shaken in an orbital shaker at 150 rpm for 15 min and, from each flask, a 10-fold dilution series in sterile 0.85% (w/v) NaCl solution was prepared. 100-*μ*L aliquots of each dilution from each series were spread onto Petri dishes containing solid (1.5% agar) YMA medium [23] with Congo red (25 mg L^−1^), vancomycin (1 *μ*g mL^−1^), and cycloheximide (55 mg L^−1^). Inoculated plates were incubated at 28°C ± 2°C for seven days. Bacterial colonies from all plates presenting between 30 and 300 colonies were counted, and the number of CFU seed^−1^ was estimated based on the average of the colony counts of the two dilutions closest to the counting range.

All experiments were completely randomized. Subsamples A and B were combined in one single replicate, so that each treatment had three replicates in the end. Results were subjected to an analysis of variance at *p* < 0,05. Further on, data were analyzed by the Dunnett (bilateral) test in order to detect significant differences between each treatment and the time zero control without chemicals, considering a 95% confidence interval. All statistical analyses were performed with the Statistica Version 7 software.

### 2.3. Field Experiments

Field experiments were conducted at sites located in representative regions of soybean growth in Brazil. The team of Embrapa Soja planted experiments in Rio Verde (17°47′S, 50°55′W; 748 m altitude; Köpen-Geiger's Aw climate) and Cachoeira Dourada (18°29′S, 49°28′W; 450 m altitude; Köpen-Geiger's Aw climate), both in the State of Goiás. Investigators at the Instituto Federal Goiano also set up experiments in Rio Verde. The investigators at Universidade Federal de Santa Catarina conducted an experiment in Curitibanos (27°16′S, 50°35′W; 995 m altitude; Köpen-Geiger's Cfa climate), in the 2013/2014 cropping season, and another in Ponte Alta do Norte (27°09′S, 50°28′W; 959 m altitude, Köpen-Geiger's Cfa climate), in the 2014/2015 cropping season, both in the State of Santa Catarina.

At all locations, samples from the soils of the experimental areas were collected 40 to 60 days prior to sowing for chemical and physical analyses performed as described before [8] and estimation of the naturalized population of soybean-nodulating bradyrhizobia by the most-probable-number method [24, 25]. The chemical, physical, and microbiological characteristics of the soils at the experimental sites are presented in Table 1. Treatments and other relevant information about the experiments are shown in Table 2.

Field plots measured at least 24 m^2^ and, in general, had eight 6 m long planting rows. Soybean seeds were sown to obtain plant populations of about 300,000 plants ha^−1^. The cultivar planted at each site is also described in Table 2. All plots were spaced by 0.5 m-wide lines and 1.5 m terraces to avoid cross contamination caused by runoff of irrigation and rainwater. Weeds, insects, and foliar diseases at all sites were controlled according to recommended agronomic practices for the soybean crop.

Plant samples (five plants) were collected from each plot during the vegetative stage, from 30 to 50 days after emergence (DAE), according to the location and weather conditions, for determination of nodule number, nodule and plant biomass, and total N in the shoots. Samples were processed in the laboratory, where roots were separated from shoots, carefully rinsed, and allowed to dry in a 50°C oven until constant weight. Nodules were then removed from the roots and allowed to dry further until they were counted and weighed. Dry shoots were weighed and ground for determination of the N content as described before [26]. Grain yield was determined at the end of the crop cycle, from the central portion (6 m^2^ to 15 m^2^) of each plot. Seeds were cleaned and weighed and grain yield was estimated after correction of seed weights to 13% moisture.

All data obtained were tested for the normality of variables and variance homogeneity, followed by an analysis of variance (ANOVA) at *p* < 0.05. In case of significance of the ANOVA, means were compared by the Duncan test, at *p* < 0.1 and *p* < 0.05, or the Tukey test at *p* < 0.05, depending on the institution that performed the experiments. Statistical software such as Sisvar, Assistat, and Statistica 7.0 was employed.

## 3. Results

### 3.1. Laboratory Experiments

The Cronos, CronosNod, and CronosTSI Technologies significantly improved the survival of the inoculated bacteria on the seeds in the presence of chemicals, when compared to the standard inoculation of untreated seeds (Table 3). On average, the Cronos and CronosNod Technologies promoted a 36-fold and a 26-fold increase, respectively, while CronosTSI promoted an astounding 366-fold increase in the number of bacteria that adhered to the treated seeds and could be recovered two hours after inoculation, compared to the standard inoculation of untreated seeds (Table 3). All products also significantly improved bacterial survival for several days after inoculation of treated seeds, and the CronosNod and CronosTSI Technologies promoted the survival of a satisfactory population of bacteria on seeds that had been treated with chemicals for up to seven and 35 days, respectively (Table 3). All technologies were, therefore, very effective in increasing the adherence of the inoculants to the seeds, promoting bacterial survival even in the presence of chemicals.

### 3.2. Field Experiments

The experiments conducted by Embrapa Soja in Rio Verde and Cachoeira Dourada suffered severe drought from sowing to early blooming, affecting early plant development and inducing great variability in the results of the parameters evaluated between 40 and 50 days after plant emergence. Nodulation was very poor at both locations and slightly superior when treated seeds received peat-based standard inoculant at sowing (Table 4). Plant biomass responded better when standard liquid inoculant was applied to untreated seeds, as did N accumulation in the shoots (Table 5). Similar results with nonsignificant differences were observed when the preinoculation technologies were employed, suggesting a positive protective effect even under adverse conditions (Table 5). The same trend could be observed for grain yield, where the preinoculation technologies promoted yields that were not significantly different from the N control or the control standard inoculants (Table 5).

In the case of the experiment conducted by the Instituto Federal Goiano, also in Rio Verde, in 2013/2014, nodulation parameters were slightly better. Preinoculation of treated seeds with all three Cronos Technologies promoted nodulation (number and biomass) that was not significantly different from the peat-based inoculant control applied at planting, but was significantly higher than either the uninoculated or the N controls, even when inoculation was performed as early as seven (CronosNod) or 35 (CronosTSI) days before planting (Table 4). Plant biomass and N accumulation in the shoots obtained with all preinoculation treatments were not significantly different from the standard inoculant and N controls, and yet significantly different from the uninoculated control (Table 5), whereas all inoculant treatments promoted significantly higher grain yields than the N and uninoculated controls (Table 5).

No treatment effects were observed on any nodulation parameters in the 2014/2015 experiment conducted by the Instituto Federal Goiano in Rio Verde (Table 4). All inoculation treatments evaluated in 2014/2015, as well as N fertilizer, however, promoted significant increases in plant biomass relative to the uninoculated control, but no significant effects were observed on N accumulation in the shoots (Table 5). All inoculation treatments produced significantly higher yields than the uninoculated control or the N fertilizer treatment (Table 5).

In Curitibanos and Ponte Alta do Norte, experiments were performed in first-year areas of soybean cropping that had been previously occupied by native pasture. Nodulation and plant parameters were also very poor, even though both nodule number and biomass tended to respond to all methods of inoculation, especially when the CronosTSI Technology was employed in Curitibanos (Table 4). No conclusive effects could be observed for plant biomass, but N accumulation in the shoots of the plants from the uninoculated and N controls and from the standard peat-based inoculant treatments was higher than that of the preinoculation treatments in Curitibanos, but not significantly different in Ponte Alta do Norte (Table 5). All treatments promoted similar grain yields, with a remarkable significant response to N fertilization in Ponte Alta do Norte (Table 4).

## 4. Discussion

Even though legumes can rely solely on N_2_ fixation to obtain all the N necessary to attain high grain yields, the effectiveness of the process depends on a fully functional set of root nodules. Nodulation is highly susceptible to abiotic and biotic factors, including the population of live bacteria on the seeds at planting [18, 27, 28]. Any factor that can cause mortality of the inoculated bacteria on the seed surface will therefore negatively affect the formation of nodules and, in turn, N_2_ fixation. Factors such as desiccation, temperature, and seed coat toxicity, for instance, have been implicated in poor inoculant survival on seeds and failure of nodulation and N_2_ fixation [7].

The utilization of industrially treated and preinoculated seeds is becoming more popular and desired among farmers, but care must be taken because many of the products employed to treat seeds are toxic, resulting in poor survival of the inoculated bacteria [21]. Agrichemicals such as fungicides applied to seeds have been shown to be very toxic to rhizobia, resulting in bacterial death just a few hours after inoculation [11, 29]. Even the conditions under which preinoculated seeds are stored can be harmful to the bacteria [30]. Therefore, if preinoculated seeds are to become widely deployed in agriculture, it is necessary to develop technologies that ensure the survival of the bacteria on the seeds for longer periods after inoculation, especially in the presence of harmful chemicals.

Many studies have addressed the issues of compatibility with agrichemicals and survival of bacteria on the surface of inoculated seeds (e.g., [21, 31]). However, in most cases, compatibility studies were performed* in vitro*, and when survival on the seeds was the issue of interest, chemicals were not always included, or the conditions under which inoculated treated seeds were stored did not mimic the real situation. Compounds such as polymers and alginate, among others, may improve the survival of bacteria [32], but little is known about their effect in the presence of chemicals. When seeds were stored under refrigeration, bacterial survival in the presence of chemicals was shown to be satisfactory [33], but there is little information about survival at room temperature.

In this study we have tested new technologies (CronosNod and CronosTSI) developed by Total Biotecnologia Indústria e Comércio S/A, which claim to favor the survival of satisfactory populations of inoculant bacteria on the surface of seeds that have been treated with a chemical (Standak Top) that combines fungicide and insecticide and were stored at room temperature. The technologies consist of a combination of a highly concentrated liquid (CronosNod) or peat-based (CronosTSI) inoculant and a protectant to be applied to seeds. The tests included the estimation of bacterial survival on treated seeds in the laboratory, and the evaluation of nodulation, plant growth, and yield under different field conditions, in comparison to standard inoculation and to another commercial preinoculation product (Cronos) of the same company that guarantees bacterial survival for up to four days on the surface of untreated seeds.

When compared to the standard peat-based inoculant, all three technologies improved the adherence of bacteria to the seed surfaces. On average, 36-, 26-, and 366-fold more bacteria could be recovered from seeds two hours after inoculation when Cronos, CronosNod, and CronosTSI were employed, respectively, in the presence of the agrichemical, when compared to the standard peat-based inoculation in the absence of agrichemicals. These results clearly demonstrate the improved adherence of the inoculants and the efficacy of all preinoculation technologies to protect the bacteria from being killed by seed treatment. The protective effect, in the presence of agrichemicals, could still be noticed two days after inoculation, when an average three (Cronos) to five (CronosNod) or even 80-fold (CronosTSI) larger population of bacteria could be recovered from treated seeds when compared to the counts obtained two hours after peat-based inoculation of untreated seeds. Even seven days after inoculation, CronosNod and CronosTSI continued to exert their protective effect, and 1.5- and 12.5-fold more bacteria, respectively, could be recovered from treated seeds than from untreated seeds that received peat-based inoculant only two hours before evaluation. CronosTSI maintained live and functional bacteria on the surface of treated seeds for as long as 35 days.

Another important aspect to be studied is the performance of the bacteria present on preinoculated seeds after planting, regarding the development of a functional set of root nodules, N_2_ fixation, and grain yield by soybean. Our study demonstrated that if seeds are to be planted right after inoculation, peat-based inoculant is most often the preferable form of inoculation. However, if preinoculation is persuaded, some protective inoculant technology must be used. Cronos, CronosNod, and CronosTSI have been shown to promote good nodulation, plant development, and grain yield, comparable to what is obtained with peat-based inoculation right before planting, when agrichemical-treated seeds were inoculated up to seven (CronosNod) or 30 days (CronosTSI) ahead of planting time.

## 5. Conclusions

CronosNod and CronosTSI are technological innovations that promote adherence to the seeds, and survival of a satisfactory population of inoculant bacteria on the surface of seeds that have received treatment with agrichemicals.

Plants grown from seeds treated with both technologies present field performances comparable to those from standard peat-based inoculants applied right before planting.

CronosNod and CronosTSI represent viable alternatives for the preinoculation of soybean seeds up to 30 days ahead of planting time.

## Figures and Tables

**Table 1:** Chemical properties, granulometry analysis, and bradyrhizobial populations of the soils at the experimental areas.

Location	Chemical properties								Granulometry			Bradyrhizobial population size^■^
	pH in CaCl_2_	Al	H + Al	K	Ca	Mg	P	C	Clay	Silt	Sand	MPN cells g^−1^
		cmol_*c*_ dm^−3^					mg dm^−3^	g dm^−3^	%			
Rio Verde^●^	5.1	0.00	3.64	0.80	1.65	1,78	9.6	22.5	36.3	9.5	54.1	zero
Rio Verde^♠^	6.1	0.03	5.10	0.65	4.40	1.30	15.2	20.2	46.4	17.4	36.2	2.50 × 10^4^
Rio Verde^♣^	5.9	0.02	4.80	0.62	4.10	1.20	14.6	18.3	46.4	17.4	36.2	3.10 × 10^4^
Cachoeira Dourada	5.4	0.00	3.07	0.37	3.65	1.73	1.7	18.5	57.7	18.2	24.0	3.57
Curitibanos	5.1	1.58	13.70	0.53	3.16	1.39	0.9	33.1	39.00	ND^*♥*^	ND	1.47 × 10^1^
Ponte Alta do Norte	4.7	1.42	7.20	0.59	0.39	0.24	0.9	20.35	ND	ND	ND	3.00 × 10^3^

^■^Most-probable-number of cells g soil^−1^. ^●^Experiment conducted by Embrapa Soja. ^♠^Experiment conducted by Instituto Federal Goiano in 2013/2014. ^♣^Experiment conducted by Instituto Federal Goiano in 2014/2015. ^*♥*^Not determined.

**Table 2:** Treatments, cultivars, numbers of replicates, and other pertinent information about field experiments.

	Rio Verde^●^	Rio Verde^♠^	Rio Verde^♣^	Cachoeira Dourada	Curitibanos	P. Alta do Norte
Treatments^■^ implanted at each location						
Uninoculated control, treated seeds	Yes	Yes	Yes	Yes	Yes	Yes
N fertilizer control, treated seeds	Yes	Yes	Yes	Yes	Yes	Yes
Standard liquid inoculant, untreated seeds	No	No	Yes	Yes	No	No
Standard peat-based inoculant, treated seeds	Yes	Yes	Yes	Yes	Yes	Yes
Standard peat-based inoculant, untreated seeds	No	No	Yes	Yes	No	No
Cronos Inoculant, two days preinoculation, treated seeds	Yes	Yes	Yes	Yes	No	Yes
Cronos Inoculant, two days preinoculation, untreated seeds	No	No	No	No	Yes	No
Cronos Inoculant, four days preinoculation, treated seeds	No	No	Yes	Yes	No	No
CronosNod Technology, seven days preinoculation, treated seeds	Yes	No	Yes	Yes	Yes	No
Cronos TSI Technology, 30 days preinoculation, treated seeds	Yes	Yes	No	No	Yes	Yes

Other relevant information						

Cultivar	NS 7209 PRO	Anta	BMX-Potência RR	BRS-GO-8360	NA 5909 (Nidera)	NA 5909 (Nidera)

Number of replicates	Four	Four	Six	Six	Five	Five

Basal fertilization	NPK 02-20-10 300 kg ha^−1^ + KCl 100 kg ha^−1^	NPK 02-20-10 300 kg ha^−1^ + KCl 100 kg ha^−1^	NPK 0-28-20 300 kg ha^−1^	NPK 0-28-20 300 kg ha^−1^	NPK 0-18-18 350 kg ha^−1^	NPK 0-18-18 350 kg ha^−1^

Micronutrients	No	No	20 g Mo ha^−1^ + 2 g Co ha^−1^, foliar spray @ V4^*♥*^	20 g Mo ha^−1^ + 2 g Co ha^−1^, foliar spray @ V4	No	No

Sampling for growth evaluation (DAE^♦^)	35	35	40	50	32	32

^■^Unless otherwise stated, seeds were treated with STANDAK TOP (see text), according to manufacturer's instructions. ^●^Experiment conducted by Instituto Federal Goiano in 2013/2014. ^♠^Experiment conducted by Instituto Federal Goiano in 2014/2015. ^♣^Experiment conducted by Embrapa Soja. ^*♥*^V4 stage: four nodes on the main stem with fully developed leaves [16]. ^♦^DAE: days after emergence.

**Table 3:** Recovery of *Bradyrhizobium *spp. cells (number of colony forming units, # CFU, per seed) from soybean seeds inoculated with different products, in the absence or presence of seed-treatment chemicals.

Treatment	Time after treatment/inoculation (duration of preinoculation)							
	# CFU seed^-1♠^							
	2 hours	2 days	4 days	7 days	14 days	21 days	28 days	35 days
	Embrapa Soja							
Standard inoculation	1.52 × 10^5^							
Cronos/on treated seeds	1.17 × 10^6^^*∗*^	7.60 × 10^5^^*∗*^	4.61 × 10^5^^*∗*^					
CronosNod on treated seeds	1.08 × 10^6^^*∗*^	8.17 × 10^5^^*∗*^	4.02 × 10^5^^ns^	1.96 × 10^5^^ns^				

	Instituto Federal Goiano							
Standard inoculation	1.83 × 10^5^							
Cronos/on treated seeds	1.24 × 10^7^^*∗*^	2.68 × 10^5^^*∗*^	1.28 × 10^5^^ns^					
CronosNod on treated seeds	8.66 × 10^6^^*∗*^	9.67 × 10^5^^*∗*^	5.20 × 10^5^^*∗*^	3.13 × 10^5^^ns^	7.70 × 10^4^	1.70 × 10^4^	6.00 × 10^3^	
CronosTSI on treated seeds	6.70 × 10^7^^*∗*^	1.40 × 10^7^^*∗*^	7.30 × 10^6^^*∗*^	2.30 × 10^6^^*∗*^	5.50 × 10^5^^*∗*^	2.40 × 10^3^	2.31 × 10^3^	1.13 × 10^3^

^♠^ *∗*  denotes a significant and ns a nonsignificant difference relative to the control, standard inoculation, evaluated two hours after inoculation, according to the bilateral Dunnett test (*p* < 0.05). All values followed by *∗* were significantly superior to the control; ns means not significantly different from control. Values not followed by *∗* or ns were not compared.

**Table 4:** Nodule number and biomass of soybean plants from the field experiments.

Treatment^■^	Nodule number (# plant^−1^)^●^						Nodule biomass (mg plant^−1^)^●^					
	RV1^♠^	RV2	RV3	CD	CB	PA	RV1	RV2	RV3	CD	CB	PA
(1)	$16.7^{\begin{array}{c} b \end{array}}$	19.8^a^	$1.07^{\begin{array}{c} d \end{array}}$	$1.37^{\begin{array}{c} ab \end{array}}$	$0.64^{\begin{array}{c} bc \end{array}}$	$0.00^{\begin{array}{c} b \end{array}}$	$116.5^{\begin{array}{c} b \end{array}}$	145.5^a^	$25.47^{\begin{array}{c} bcd \end{array}}$	14.8^a^	$3.56^{\begin{array}{c} ab \end{array}}$	$0.00^{\begin{array}{c} b \end{array}}$
(2)	15.1^b^	16.2^a^	0.23^d^	0.17^b^	0.40^c^	0.04^b^	105.5^b^	115.5^ab^	4.11^d^	0.4^b^	0.58^b^	0.13^b^
(3)	29.7^a^	17.1^a^	21.53^a^	2.67^a^	1.24^ab^	1.76^a^	207.5^a^	87.5^b^	108.69^a^	18.5^a^	5.85^ab^	2.53^a^
(4)	NA^*♣*^	NA	0.73^d^	2.20^a^	NA	NA	NA	NA	17.05^cd^	20.0^a^	NA	NA
(5)	NA	NA	NA	NA	1.48^a^	NA	NA	NA	NA	NA	5.40^ab^	NA
(6)	28.2^a^	17.9^a^	3.57^bc^	2.33^a^	NA	0.32^b^	198.2^a^	96.0^b^	48.39^bc^	19.9^a^	NA	0.12^b^
(7)	NA	NA	5.23^b^	1.97^a^	NA	NA	NA	NA	63.45^b^	18.7^a^	NA	NA
(8)	29.3^a^	NA	2.10^cd^	1.83^a^	1.56^a^	NA	205.6^a^	NA	28.4^bcd^	15.6^a^	8.00^a^	NA
(9)	28.4^a^	14.4^a^	NA	NA	3.76^a^	0.20^b^	199.6^a^	103.0^ab^	NA	NA	13.06^a^	1.18^a^

*p* value	<0.05	<0.05	<0.1	<0.1	<0.05	<0.05	<0.05	<0.05	<0.1	<0.1	<0.05	<0.05

^■^Treatment descriptions: (1) Uninoculated, not N-fertilized control, treated seeds; (2) N-fertilized control, treated seeds; (3) Standard peat-based inoculant applied on planting day, treated seeds; (4) Standard liquid inoculant applied on planting day, untreated seeds; (5) Cronos inoculant, two days pre-inoculation, untreated seeds; (6) Cronos inoculant, two days pre-inoculation, treated seeds; (7) Cronos inoculant, four days pre-inoculation, treated seeds; (8) CronosNod technology, seven days pre-inoculation, treated seeds; (9) CronosTSI technology, 30 days pre-inoculation, treated seeds. ^●^Means followed by the same letter, on the same column, are not significantly different, according to the comparison tests described in the Materials and Methods section. ^♠^RV1 – experiment conducted in Rio Verde by Instituto Federal Goiano in 2013/2014; RV2 - experiment conducted in Rio Verde by Instituto Federal Goiano in 2014/2015, RV3 – experiment conducted in Rio Verde by Embrapa Soja, CD – experiment conducted in Cachoeira Dourada by Embrapa Soja, CB – experiment conducted in Curitibanos by Universidade Federal de Santa Catarina; PA – experiment conducted in Ponte Alta do Norte by Universidade Federal de Santa Catarina. ^♣^NA = not applicable; the treatment was not included at the specific experimental site.

**Table 5:** Plant biomass, N accumulation in the shoots, and grain yield of soybean plants from the field experiments.

Treatment^■^	Plant biomass (g plant^−1^)^●^						N accumulation (mg N plant^−1^)^●^						Grain yield (kg ha^−1^)^●^					
	RV1^♠^	RV2	RV3	CD	CB	PA	RV1	RV2	RV3	CD	CB	PA	RV1	RV2	RV3	CD	CB	PA
(1)	$5.0^{\begin{array}{c} b \end{array}}$	$4.7^{\begin{array}{c} b \end{array}}$	$6.92^{\begin{array}{c} a \end{array}}$	$9.45^{\begin{array}{c} a \end{array}}$	$2.69^{\begin{array}{c} ab \end{array}}$	$5.34^{\begin{array}{c} a \end{array}}$	$108.0^{\begin{array}{c} b \end{array}}$	$107.5^{\begin{array}{c} a \end{array}}$	$153.9^{\begin{array}{c} abc \end{array}}$	$228.5^{\begin{array}{c} ab \end{array}}$	$87.8^{\begin{array}{c} ab \end{array}}$	$33.5^{\begin{array}{c} a \end{array}}$	$2207^{\begin{array}{c} b \end{array}}$	$1916^{\begin{array}{c} c \end{array}}$	$2552^{\begin{array}{c} b \end{array}}$	$2717^{\begin{array}{c} abc \end{array}}$	$3195^{\begin{array}{c} b \end{array}}$	2731^c^
(2)	6.5^ab^	7.1^a^	6.53^ab^	8.41^ab^	2.69^ab^	5.39^a^	115.0^a^	109.0^a^	200.1^a^	270.3^a^	85.6^ab^	34.4^a^	2181^b^	2337^bc^	2855^a^	2531^bc^	3873^ab^	4445^a^
(3)	6.5^ab^	7.4^a^	6.46^ab^	7.97^ab^	3.34^a^	5.19^a^	111.0^ab^	108.5^a^	174.2^abc^	229.1^ab^	89.2^a^	36.3^a^	3833^a^	3134^a^	3050^a^	2658^abc^	3808^ab^	2905^b^
(4)	NA^*♣*^	NA	7.86^a^	9.51^a^	NA	NA	NA	NA	196.1^ab^	276.2^a^	NA	NA	NA	NA	2586^b^	2477^c^	NA	NA
(5)	NA	NA	NA	NA	2.24^b^	NA	NA	NA	NA	NA	60.7^c^	NA	NA	NA	NA	NA	3956^a^	NA
(6)	7.0^a^	7.2^a^	5.34^b^	8.93^ab^	NA	5.43^a^	113.0^a^	109.0^a^	135.4^c^	248.7^ab^	NA	35.3^a^	3745^a^	3189^a^	2893^a^	2880^ab^	NA	3276^b^
(7)	NA	NA	7.55^a^	7.47^ab^	NA	NA	NA	NA	200.5^a^	179.7^b^	NA	NA	NA	NA	2807^a^	2701^abc^	NA	NA
(8)	7.0^a^	NA	6.37^ab^	7.11^b^	2.65^ab^	NA	114.0^a^	NA	148.8^bc^	172.8^b^	68.1^bc^	NA	3696^a^	NA	2895^a^	2950^a^	3479^ab^	NA
(9)	6.3^a^	8.1^a^	NA	NA	3.02^a^	5.91^a^	114.0^a^	104.5^a^	NA	NA	66.4^bc^	36.0^a^	3594^a^	2977^a^	NA	NA	3783^a^	3023^b^

*p* value	<0.05	<0.05	<0.1	<0.1	<0.05	<0.05	<0.05	<0.05	<0.1	<0.1	<0.05	<0.05	<0.05	<0.05	<0.1	<0.1	<0.05	<0.05

^■^Treatment descriptions: (1) uninoculated, not N-fertilized control, treated seeds; (2) N-fertilized control, treated seeds; (3) standard peat-based inoculant applied on planting day, treated seeds; (4) standard liquid inoculant applied on planting day, untreated seeds; (5) Cronos inoculant, two days preinoculation, untreated seeds; (6) Cronos inoculant, two days preinoculation, treated seeds; (7) Cronos inoculant, four days preinoculation, treated seeds; (8) CronosNod technology, seven days preinoculation, treated seeds; (9) CronosTSI technology, 30 days preinoculation, treated seeds. ^●^Means followed by the same letter, on the same column, are not significantly different, according to the comparison tests described in the Materials and Methods. ^♠^RV1: experiment conducted in Rio Verde by Instituto Federal Goiano in 2013/2014; RV2: experiment conducted in Rio Verde by Instituto Federal Goiano in 2014/2015, RV3: experiment conducted in Rio Verde by Embrapa Soja, CD: experiment conducted in Cachoeira Dourada by Embrapa Soja, CB: experiment conducted in Curitibanos by Universidade Federal de Santa Catarina; PA: experiment conducted in Ponte Alta do Norte by Universidade Federal de Santa Catarina. ^♣^NA = not applicable; the treatment was not included at the specific experimental site.
